# Expanding the Phenotypic Spectrum of Differentiated High-Grade Thyroid Carcinoma: An Extracranial Mass as the First Presentation

**DOI:** 10.1155/crie/1165861

**Published:** 2025-10-12

**Authors:** Mennaallah Eid, Michael Barndon Stone, Tibor Valyi-Nagy

**Affiliations:** ^1^Division of Endocrinology, Diabetes and Metabolism, University of Illinois at Chicago, Chicago, Illinois, USA; ^2^Department of Pathology, University of Illinois at Chicago, Chicago, Illinois, USA

## Abstract

**Background:**

Differentiated high-grade thyroid carcinoma (DHGTC) is a recently recognized entity introduced in the 2022 World Health Organization (WHO) Classification of Endocrine and Neuroendocrine Tumors. The prognosis is intermediate between differentiated thyroid carcinoma, which generally has a favorable outcome, and anaplastic thyroid carcinoma, which carries a poor prognosis. Given its recent classification, standardized management guidelines and long-term follow-up data are lacking.

**Case Presentation:**

We report a case of a 52-year-old male who presented with a protruding cranial mass with overlying wound dehiscence. Initial pathological analysis of the mass revealed metastatic thyroid carcinoma. Further workup identified a 7 cm × 5 cm mass at the right vertex, invading the right parietal bone and extending into the epidural space. Additionally, a large thyroid mass was observed, causing destruction of the manubrium sternum and upper ribs, with subcutaneous and superior mediastinal extension. The patient underwent cranial mass resection and biopsy of the superior mediastinal mass. Histopathological examination confirmed DHGTC with papillary features and tall cell morphology, involving the dura mater, parietal bone, and scalp. The superior mediastinal biopsy also demonstrated high-grade thyroid carcinoma. The patient had a history of autism spectrum disorder with nonverbal communication. Given the advanced disease stage, the family opted for comfort care.

**Conclusion:**

This case highlights the new histopathological criteria for DHGTC and its unusual presentation, emphasizing the challenges in management and prognosis of this newly defined entity. Clinicians should consider DHGTC in atypical extracranial metastatic presentations to facilitate early diagnosis and multidisciplinary management.

## 1. Introduction

Differentiated high-grade thyroid carcinoma (DHGTC) is a newly classified thyroid carcinoma with aggressive features and a worse prognosis than conventional differentiated thyroid cancer (DTC). Cranial bone metastasis as an initial presentation of DHGTC is extremely rare. We present a case of DHGTC initially manifesting as a protruding cranial mass, highlighting diagnostic and therapeutic challenges.

The classification of thyroid carcinoma has evolved based on histopathological characteristics, leading to changes in management and prognosis. The 2017 World Health Organization (WHO) Classification of Endocrine and Neuroendocrine Tumors categorized follicular cells derived thyroid carcinomas into papillary, follicular, oncocytic thyroid carcinoma, and poorly differentiated thyroid carcinoma (PDTC) [1]. However, a distinct subset of well-DTC cells exhibiting high mitotic activity and/or tissue necrosis was identified as having a poor prognosis, regardless of cellular differentiation. This discovery prompted an update in the 2022 WHO Classification, introducing DHGTC as a new entity alongside PDTC under the high-grade follicular cell-derived thyroid carcinomas category [2]. While most DTCs are associated with a favorable prognosis, high survival rates, and low metastatic potential, DHGTC exhibits a worse prognosis, higher recurrence rates, and an increased likelihood of distant metastases [3].

## 2. Case Presentation

A 52-year-old male with a history of autism spectrum disorder and coronary artery disease presented to another facility with a progressively enlarging cranial mass for 1 month. An attempted subcutaneous aspiration under local anesthesia was unsuccessful, complicated by bleeding and extravasation, yielding only a few aspirated cells. Hemostasis was achieved with sutures, and the patient was subsequently transferred to our center. Pathological examination of the aspirated sample revealed metastatic thyroid cancer cells. On physical examination, the patient was hemodynamically stable, afebrile, and in no apparent distress. As per his baseline, he was nonverbal, with no new neurological deficits. There were no signs of increased intracranial pressure such as papilledema, nausea, or altered mental status. The patient was neurologically intact on examination. Head examination revealed a 6 cm × 5 cm mass at the right vertex, protruding through the scalp with dehiscence and bloody discharge. Additionally, a diffuse neck fullness and a palpable 4 cm × 5 cm subcutaneous mass over the manubrium sternum were noted. There were no signs of vascular occlusion or facial edema. Laboratory findings showed normal complete blood count, liver and kidney function tests, and thyroid-stimulating hormone levels.

**Figure 1:**
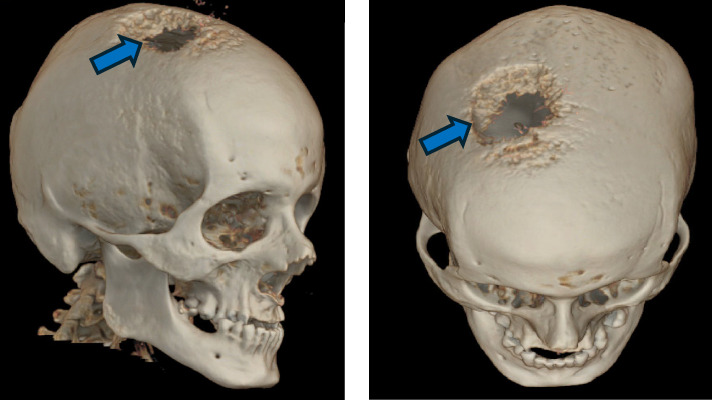
Three dimension computed tomography of the head shows a right parietal bone destruction (blue arrow) by metastasis from differentiated high grade thyroid carcinoma. The metastasis destroyed the parietal bone, invaded the dura and scalp and protruded extracranially.

**Figure 2:**
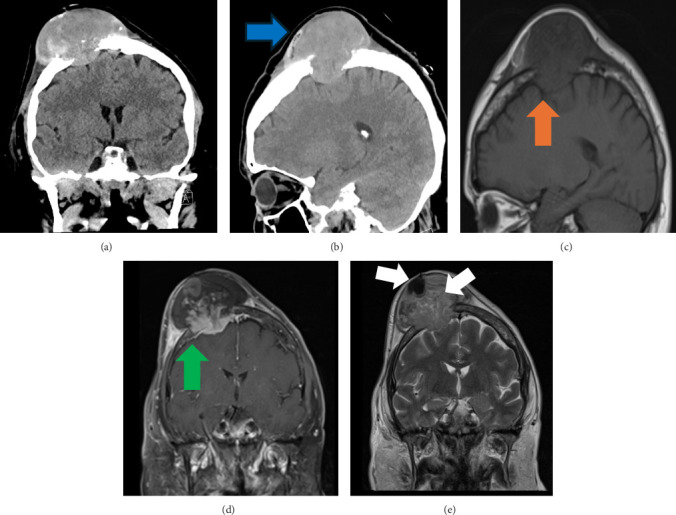
Computed tomography of the head. (A) Coronal view and (B) sagittal view. Brain magnetic resonance imaging: (C) sagittal T1 view, (D) coronal T1 view, and (E) coronal T2 view. Imaging showed a right paramedian parietal protruding mass (blue arrow) destroying the skull and extending to the epidural subdural space (green arrow) with localized mass effect (orange arrow) and internal calcification and necrosis (white arrow).

**Figure 3:**
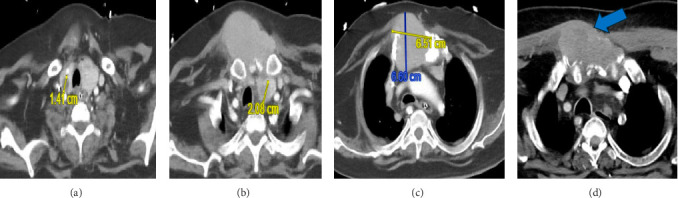
Computed tomography of the chest in axial view showed thyroid enlargement with right thyroid nodule (A) and left thyroid nodule (B) and superior mediastinal mass (C) and subcutaneous extension (blue arrow) and destruction of manubrium sternum (D).

**Figure 4:**
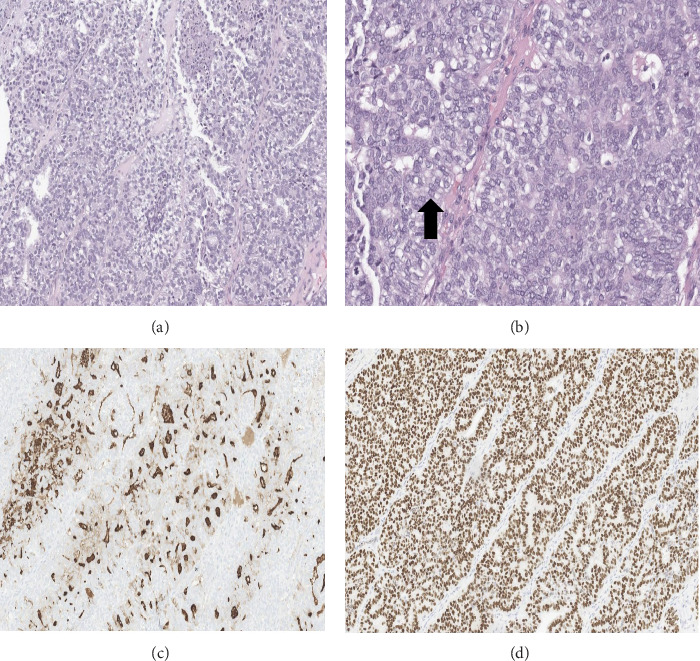
Histopathology of the resected head mass showed differentiated high-grade thyroid carcinoma with invasion of the scalp, dura, and parietal bone. (A) H&E 100x magnification and (B) H&E 200x magnification. Malignant thyroid cells with papillary features, high mitotic rate, and foci of necrosis and tall cell features (black arrow). (C) Tumor cells with thyroglobulin expression (100x magnification thyroglobulin immunohistochemistry). (D) Tumor cells with nuclear expression of TTF-1 (100x magnification TTF-1 immunohistochemistry).

Head computed tomography (CT scan, coronal view) showed a 7.5 cm × 7.2 cm × 5.5 cm mass at the right vertex, with calcifications, heterogeneous enhancement, and destruction of the underlying right parietal bone (Figures 1 and 2). Brain magnetic resonance imaging (MRI, coronal view) revealed a right paramedian parietal mass (6.9 cm × 5.7 cm × 5.2 cm) with significant extracranial extension into the scalp and extra-axial involvement of the epidural and subdural spaces (Figure 2). There were no imaging signs of increased intracranial pressure, suggesting compensated intracranial pressure at presentation. Neck and chest CT scans demonstrated an extensive bony destruction of the manubrium sternum and proximal first ribs and a 6.5 cm × 6.6 cm mass extending into the subcutaneous soft tissue and anterior superior mediastinum (Figure 3). Thyroid gland enlargement with bilateral hypoattenuating nodules (1.4 cm in the right lobe, 2 cm in the left; Figure 3). No lymphadenopathy or distant organ involvement on CT scans of the neck, abdomen, or pelvis. The patient underwent craniotomy with resection of the scalp, dura mater, and parietal bone overlying the mass. The calvarium appeared mottled and infiltrated with cancerous tissue beyond the mass margins, necessitating a biparietal craniotomy. The underlying brain parenchyma was pulsatile and healthy. A gross-total resection of the mass was achieved, and cranioplasty was performed in the same setting. Histopathology revealed malignant thyroid cancer cells with papillary thyroid carcinoma (PTC) nuclear features, a high mitotic index (10–12 mitotic figures per 2-mm^2^), and a prominent tall-cell morphology. The resected specimen showed extension of the tumor into the parietal bone, periosteum, dura mater, and scalp without involvement of epidermal layer (Figure 4). Immunohistochemistry studies were positive for cytokeratin CAM5.2, thyroid transcription factor 1 (TTF-1), and thyroglobulin and negative for chromogranin and synaptophysin. The tumor was classified as DHGTC with skull, meningeal, and scalp metastases. A biopsy of the sternal mass was performed under interventional radiology guidance, revealing tumor cells with areas of necrosis. Immunohistochemistry study was positive for CAM5.2, CK AE1/AE3, TTF-1, and PAX8 and negative for chromogranin, synaptophysin, thyroglobulin, calcitonin, napsin A, and GATA3. Few cells expressed p63. Findings confirmed DHGTC with sternal metastasis. The patient's goals of care, prognosis, and survival expectations were discussed with the family. Given the advanced disease stage, the family opted for comfort care and hospice referral.  Table 1 summarizes the key clinical events.

## 3. Discussion

DTC is the most common follicular cell-derived thyroid cancer, generally associated with a favorable prognosis and high survival rates. However, DHGTC is a newly recognized and rare subtype with a significantly poorer prognosis, despite its well-differentiated histological features [3].

Cranial bone metastases are rarely observed in DTC and are typically reported in association with follicular or papillary subtypes, often appearing years after the initial diagnosis of the primary tumor. The most frequently involved distant metastatic sites in DHGTC include the lungs, axial skeleton, and mediastinum, with cranial involvement remaining exceptionally uncommon [3, 4]. While sporadic case reports have described skull metastases from PTC—some involving the dura or venous sinuses—these have uniformly occurred in the setting of previously diagnosed thyroid malignancy, not as an initial presentation [5, 6]. Cranial bone metastases may spread through several mechanisms, including hematogenous dissemination via arterial circulation, retrograde venous spread through the valveless Batson's plexus, direct extension from adjacent structures, and, less commonly, perineural spread along cranial nerves [6].

To date, there are no documented cases in the literature describing cranial bone metastasis with epidural extension as the first manifestation of DHGTC, underscoring the rarity and clinical significance of our case. We report a case of DHGTC with extensive local invasion and distant metastases, involving the skull, dura mater, scalp, and sternum.

The patient initially presented with a large, protruding cranial mass, later identified as metastatic thyroid carcinoma with invasion of the parietal bone and dura mater. Physical examination was remarkable for full neck and subcutaneous swelling over the manubrium sternum. Additional findings included thyroid enlargement with two nodules, superior mediastinal and subcutaneous extension, and involvement of the manubrium sternum and ribs. The patient underwent a biparietal craniotomy with complete resection of the cranial mass, and a biopsy of the mediastinal mass confirmed DHGTC. Notably, despite the extensive disease burden, the patient had no new neurological deficits or problems swallowing and breathing difficulty from the thyroid enlargement and local extension.

Our initial treatment plan included total thyroidectomy and resection of the mediastinal mass, followed by molecular testing to guide adjuvant therapy. Potential therapeutic approaches included radioactive iodine therapy (if iodine-avid), external beam radiation therapy (EBRT), immunotherapy, and antiresorptive therapy (e.g., bisphosphonates or denosumab) for bone metastases. However, after discussing the prognosis and expected survival rate, the patient's family preferred comfort care and hospice referral, and preventing further invasive interventions or molecular testing.

DHGTC is defined as a well-differentiated follicular cell-derived thyroid carcinoma with a high mitotic index (≥5 mitoses per 2-mm^2^) and/or tumor necrosis. The most common histological subtype is PTC, followed by follicular and oncocytic variants [2]. PDTC was first described in 1983 [7] and later refined in 2007 [8]. PDTC is characterized by solid, trabecular, and insular growth patterns, absence of nuclear features of DTC, and either convoluted nuclei, a mitotic index of ≥3 mitoses per 2 mm^2^, or tumor necrosis [8]. Both DHGTC and PDTC are classified as high-grade follicular cell-derived thyroid carcinomas without anaplastic dedifferentiation, according to the 2022 WHO Classification [2].

DHGTC and PDTC are more common in females over 50 years of age, with an estimated prevalence of <5% of thyroid cancers in the USA [9, 10]. In one single-center study, DHGTC accounted for 1.3% of DTC cases, with PTC and a predominant tall-cell component being the most common histological subtype which is consistent with our case. Moreover, in this study, DHGTC tumors tended to be larger, exhibited increased vascular invasion, extrathyroidal extension, and distant metastases at diagnosis [3]. A study of 32 DHGTC cases reported an average age of 52.6 years and a higher incidence in males with predominancy of PTC with a classic subtype like our case [12].

Both PDTC and DHGTC typically express TTF-1, PAX8, cytokeratin (e.g., CK7), and thyroglobulin, with a Ki-67 proliferation index ranging between 10% and 30% [2]. In our case, the cranial metastasis stained positive for cytokeratin CAM5.2, TTF-1, and thyroglobulin and the sternal biopsy stained positive for CAM5.2, CK AE1/AE3, TTF-1, and PAX8, but negative for thyroglobulin. The absence of thyroglobulin staining could be due to weak thyroglobulin expression, which has been reported in this subset of thyroid carcinomas [2]. p63, a marker occasionally found in PTC and Hashimoto's thyroiditis, was detected, though its prognostic significance remains unclear [11]. Negative staining for chromogranin and synaptophysin ruled out medullary thyroid carcinoma.

Studies have found that the molecular testing studies suggest RAS mutations are more common in PDTC, whereas BRAF V600E mutations are more frequently observed in DHGTC [10]. Additionally, DHGTC is associated with a higher frequency of telomerase reverse transcriptase (TERT) promoter mutations compared to conventional DTC, contributing to its aggressive clinical behavior [3].

The prognosis of DHGTC is poor, comparable to DTC, with an increased risk of recurrence and distant metastases, particularly in cases with extensive tumor necrosis [3]. The survival rates for PDTC are 89%, 76%, 60%, and 35% at 3, 5, 10, and 20 years, respectively, and similar rates have been observed for high-grade thyroid carcinomas not meeting PDTC criteria (including DHGTC) [9, 10, 12]. However, other studies suggest DHGTC may have even lower survival rates than PDTC [13]. Poor prognostic factors include older age, extensive local invasion, distant metastases, and extensive tumor necrosis [10].

The management of DHGTC and PDTC is not standardized, and responses to conventional DTC therapies are highly variable. Approximately 50% of cases are resistant to radioactive iodine therapy [2]. The general treatment approach includes total thyroidectomy with or without neck dissection followed adjuvant therapies as radioactive iodine therapy, if iodine-avid (though up to 50% are resistant) [2] or EBRT for non-iodine-avid or locally advanced disease. Systemic therapies including tyrosine kinase inhibitors (TKIs): Lenvatinib and Sorafenib; BRAF-targeted therapy (for BRAF V600E mutation): Dabrafenib and Trametinib; RET-targeted therapy (for RET mutations): Selpercatinib and Pralsetinib [10]. Antiresorptive therapy is recommended for skeletal metastases. In our case, further molecular and genetic testing could not be pursued, as the patient's family did not prefer additional investigations and preferred comfort care.

## 4. Limitations

This case is limited by the absence of molecular profiling and lack of long-term outcome data due to palliative decision-making.

## 5. Conclusion

DHGTC is a newly recognized and aggressive thyroid carcinoma with a poor prognosis and higher rates of recurrence and distant metastases. Early recognition of DHGTC is critical due to its aggressive behavior and atypical metastatic potential. A multidisciplinary approach is essential to ensure timely diagnosis, individualized treatment planning, and improved patient outcomes.

## Figures and Tables

**Table 1:** A timeline table summarizing key clinical events.

Date/time frame	Clinical event
Approximately 1 month before admission	Onset of protruding cranial mass
Day 1	Subcutaneous aspiration attempted at referring facility (unsuccessful, with bleeding)
Day 2–3	Transfer to our center
Day 4	Initial evaluation and imaging (CT/MRI showing parietal bone destruction and mediastinal mass)
Day 5–6	Pathology confirmed metastatic thyroid carcinoma on aspiration sample
Day 7	Craniotomy with resection of cranial mass and cranioplasty
Day 10	Biopsy of sternal/superior mediastinal mass under interventional radiology
Day 11–12	Histopathology confirmed DHGTC in both cranial and mediastinal sites
Day 13	Prognosis discussion with family
Day 14	Decision for palliative care and hospice referral

## Data Availability

All data generated or analyzed during this study are included in this published article. Further inquiries can be directed to the corresponding author.
